# Supplementary material for: Antioxidant and Chemopreventive Activity of Protein Hydrolysates from Raw and Germinated Flour of Legumes with Commercial Interest in Colorectal Cancer
Source: Antioxidants (Basel). 2022 Dec 8;11(12):2421. doi: 10.3390/antiox11122421 (PMC9774143; doi:10.3390/antiox11122421)
Supplement: Supplementary file 1 [file antioxidants-11-02421-s001.zip › Figure S1.pdf]

SUPPLEMENTARY MATERIAL

Antioxidant and chemopreventive activity of protein hydrolysates from raw and germinated flour of legumes with commercial interest in colorrectal cancer

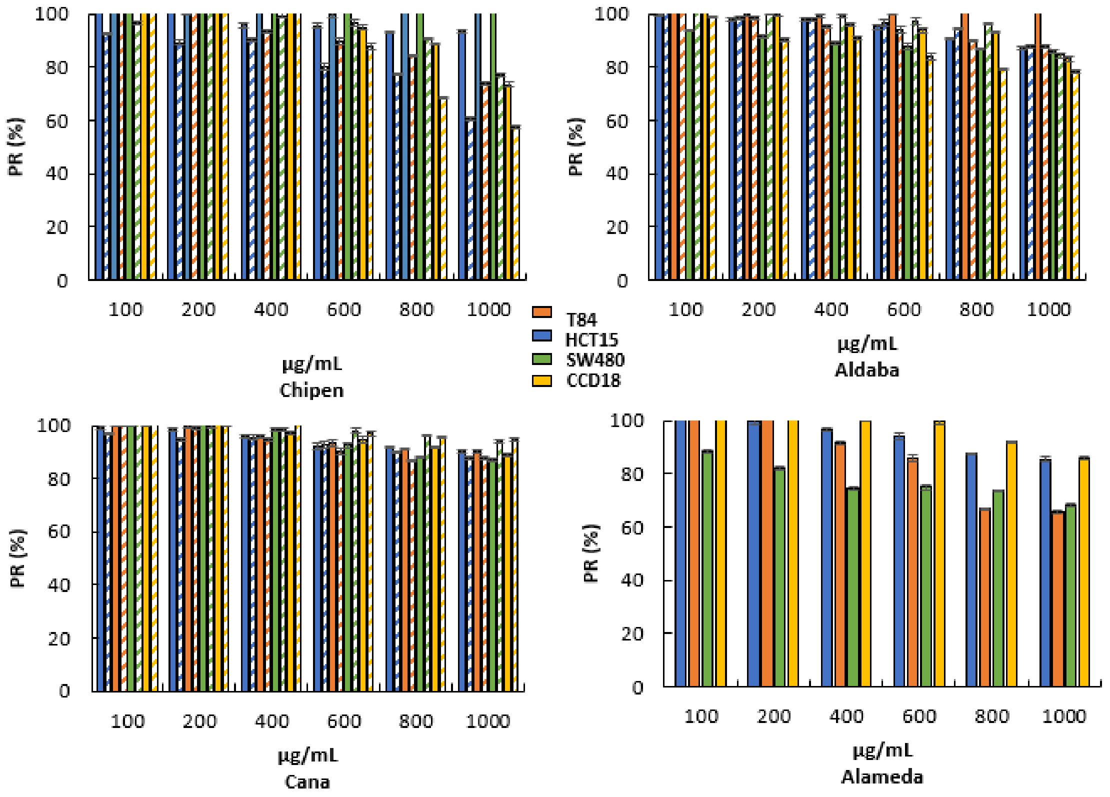

Figure S1. Graphic representation of the percentage of relative proliferation (PR (%)) in different cell lines of the varieties of raw (solid columns) and germinated (striped columns) legumes in protein hydrolysates (B).
